# Supplementary material for: Attosecond spectroscopy reveals alignment dependent core-hole dynamics in the ICl molecule
Source: Nat Commun. 2020 Nov 16;11:5810. doi: 10.1038/s41467-020-19496-0 (PMC7669856; doi:10.1038/s41467-020-19496-0)
Supplement: Supplementary file 1 — Supplementary Information [file 41467_2020_19496_MOESM1_ESM.pdf]

Supplementary material: Attosecond spectroscopy reveals alignment dependent core-hole dynamics in the ICl molecule.

Authors:

Hugo J. B. Marroux,[1,2,†,*] Ashley P. Fidler,[1,2] Aryya Ghosh,[4] Yuki Kobayashi,[1] Kirill Gokhberg,[4] Alexander I. Kuleff,[4,5] Stephen R. Leone,[1,2,3*] Daniel M. Neumark[1,2*]

Affiliations:

[1]Department of Chemistry, University of California, Berkeley, 94720, USA.
[2]Chemical Sciences Division, Lawrence Berkeley National Laboratory, Berkeley, 94720, USA.
[3]Department of Physics, University of California, Berkeley, 94720, USA
[4]Theoretische Chemie, PCI, Universität Heidelberg, Im Neuenheimer Feld 229, 69120, Heidelberg, Germany
[5]ELI-ALPS, W. Sandner utca 3, 6728 Szeged, Hungary
*Correspondence to: hugo.marroux@epfl.ch; srl@berkeley.edu; dneumark@berkeley.edu
†Current address: Laboratoire de Spectroscopie Ultrarapide (LSU) and Lausanne Centre for Ultrafast Science (LACUS), Ecole Polytechnique Fédérale de Lausanne, ISIC, FSB, Station 6, CH-1015 Lausanne, Switzerland

SM1: Static absorption spectrum fitting

In the core to Rydberg transitions considered here, the core-excited state nuclear potential is bound, as it results from the transfer of an electron from a non-bonding orbital (the core 4*d* level) to a non-bonding 6*p* Rydberg orbital.[1] This is confirmed by computing electronic potential energy curves for the ten $4d^{-1}6p$ states obtained considering the five core-hole orbitals and the two $6p\sigma$ and $6p\pi$ orbitals as shown in Fig. S1a.

Computation of the core-excited potentials of ICl was performed by using the spin-orbit generalized multiconfigurational perturbation theory.[1,2] The ZFK-DK3 relativistic model core potentials and basis sets of triple-zeta quality are used.[3] A Hartree-Fock self-consistent field computation (SCF) is performed at $R = 2.32$ Å and the resultant molecular orbitals (MOs) are used as initial orbitals for the subsequent multiconfigurational self-consistent field (MCSCF) calculations. We performed two calculations separately. In one set of calculations, electronic configurations of $(\text{I-}4d)^{10}(\sigma^*)^0(6p_{x,y})^0$, $(\text{I-}4d)^9(\sigma^*)^1(6p_{x,y})^0$, $(\text{I-}4d)^9(\sigma^*)^0(6p_{x,y})^1$ are included. In the other set of calculations, electronic configurations of $(\text{I-}4d)^{10}(\sigma^*)^0(6s6p_z)^0$, $(\text{I-}4d)^9(\sigma^*)^1(6s6p_z)^0$, $(\text{I-}4d)^9(\sigma^*)^0(6s6p_z)^1$ are included. In order to obtain accurate spin-orbit splitting for the I-4*d* shell, the effective nuclear charge of $Z_{\text{eff}} = 72.0$ was used for the iodine atom.

Computation of the core-excited state potential energy curves were done using the SO-GMC-QDPT code[2] implemented in the developer version of the GAMESS-US program package.[4] The ZFK-DK3 relativistic model core potential (MCP) and basis sets of triple-zeta quality[5] are used. A Hartree-Fock self-consistent field (SCF) computation is performed at the ground-state equilibrium internuclear distance of $R_{\text{equ}} = 2.3$ Å. The resultant molecular orbitals are used as initial orbitals for the subsequent state-averaged multi-configurational self-consistent field (SA-MCSCF) computations.

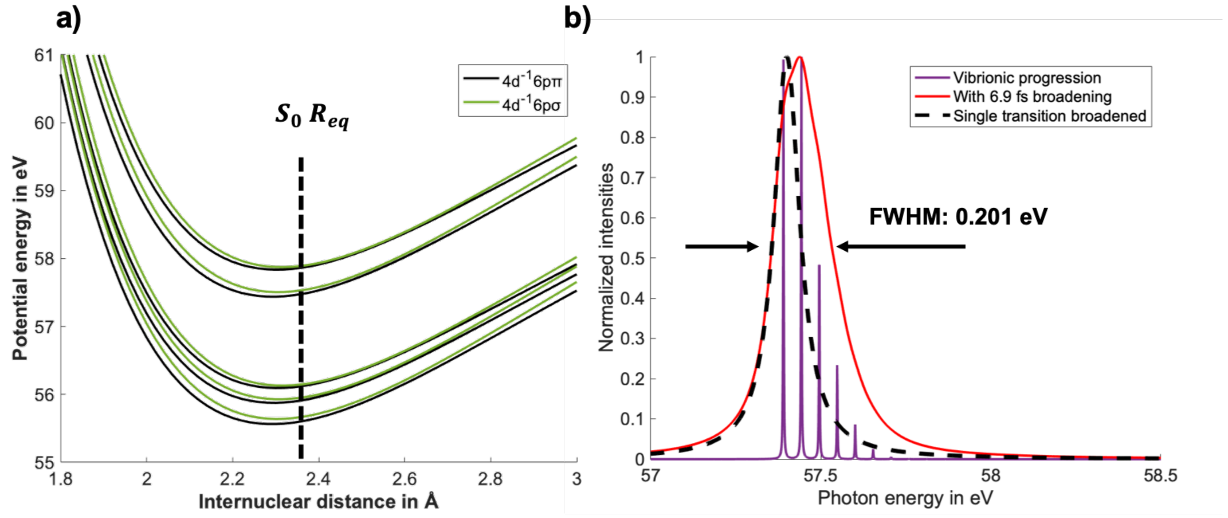

Figure S1 a) Potential energy curves computed for the core-excited $4d^{-1}6p$ states of ICl. States resulting from transitions to a 6pσ and 6pπ orbitals are shown in green and black, respectively. **b)** In purple, vibronic progression from v=0 considering two displaced harmonic oscillators (cf. text for details) for the CES with $\Delta_{3/2}$ core-hole character. In dashed black, a single vibronic transition broadened by a lifetime of 6.9 fs and in solid red, absorption feature resulting from broadening the entire vibronic progression with a lifetime of 6.9 fs.

Upon excitation of the core level, the equilibrium internuclear distance shortens by 0.07 Å on average and the vibrational frequency of ICl increases from 385 cm^{-1} to 420-430 cm^{-1}. The spectrum in purple shown in Fig. S1b is the vibronic progression obtained by computing the Franck-Condon overlap considering displaced harmonic oscillators for the $\Delta_{3/2}$ core-hole using the ezSpectrum software suite.[6] Considering the lifetime measured for that state by ATAS (6.9 fs or 0.095 eV) a single vibronic transition is broadened to obtained the spectrum shown as a dashed black line in Fig. S1b while the vibronic progressions merge into a single feature shown with a solid red line with a full width at half maximum of 0.201 eV.

To obtain the fit to the absorption spectrum of Fig. 1b in the main text, the calculation of vibronic progression is repeated for the four excited states convoluted with a 50 meV spectral resolution. The four features are then fit to the static absorption spectrum by varying values for the lifetimes, positions and intensities. Central positions are obtained with an error <10 meV and used to extract kinetic traces discussed in the main text and SM2, and lifetimes are shown in Table S1.

Table S1 *Lifetime obtained from lineshape analysis*

Energy (eV)	55.6	55.9	57.4	57.7
Lifetime from static spectrum fit	6.1±1.8 fs	4.4±1.1 fs	7.7±2.1 fs	5.1±1.3 fs

At first glance, the lifetime variation with core-hole orbital alignment seems to be captured by the frequency domain analysis, but error bars in the obtained lifetime values are too large to accurately interpret the alignment effect based only on the lineshape analysis.

SM2: Feature overlap

In the static spectrum of ICl, features in the $4d^{-1}6p$ manifold overlap, leading to potential bias in the dephasing times measured. The kinetics traces obtained at peak central frequencies (obtained from fitting of the static absorption spectrum presented in SM1) are shown as dots in Fig. S2a and S2b (reproduced from Fig. 2b and 2c in the main text). The traces are fitted by the convolution of a Heaviside (to enforce causality), an exponential decay and a Gaussian function. Error bars on the kinetic fits obtained are between 5 and 11%.

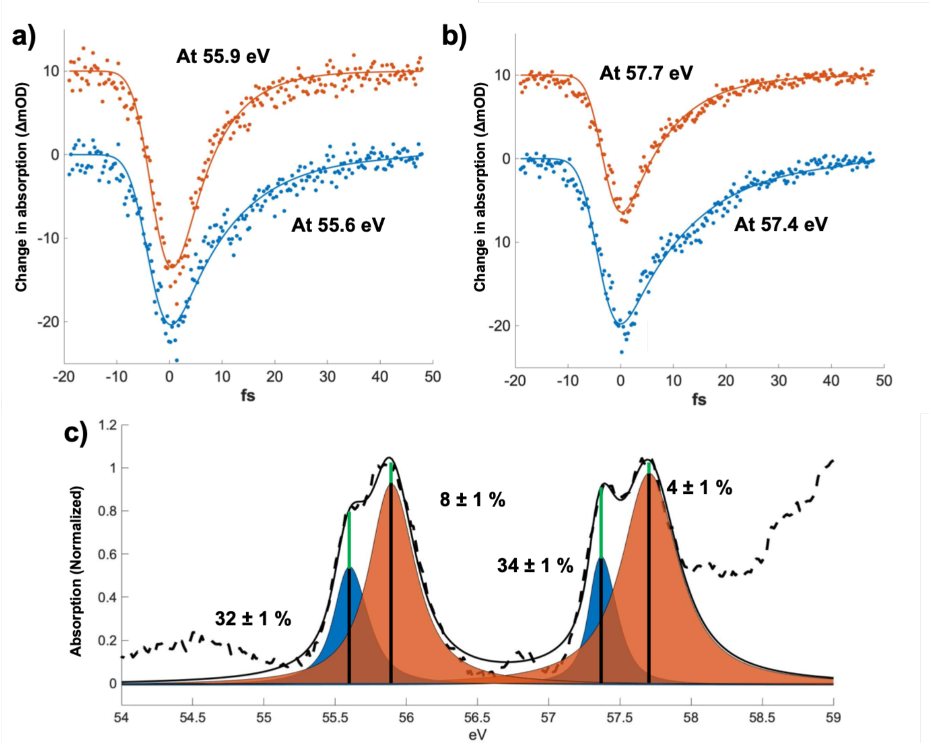

Figure S2a) and **b):** Kinetics traces and exponential decay fits (cf. text for detail) at the indicated energies. **c)** Experimental static spectrum (dashed line) and fit (solid line) using the four transitions in blue and orange (cf. text for details). The black and green stick represent the relative main and overlapping contributions at each energy.

Fig S2c shows the decomposition of the static absorption spectrum. The energies for the measured kinetic traces are indicated by the black and green sticks. The black stick lengths are proportional to the contribution of the main peaks at each frequency and the green stick lengths are proportional to the intensities of the nearby peaks. The percentages shown in Fig. S2c are the resulting overlap contributions. Table S2 summarizes the noted error bars obtained from the kinetic fits and the overlap contributions from Fig. S2c.

Table S2: *Kinetic fit time constants and relative errors at the indicated energies with overlaps contaminations*

Probed energy	**55.6 eV**	**55.9 eV**	**57.4 eV**	**57.7 eV**

Fit time constants	11.0±0.8 fs	7.0±0.8 fs	13.2±0.6 fs	8.6±0.6 fs
Kinetics fits error	±7 %	±11 %	±5 %	±7 %
Overlap	32 ± 1 %	8 ± 1 %	34 ± 1 %	4 ± 1 %

The relative errors of the kinetic fits, at the energies of the orange peaks, ($\Omega_c = 3/2,\ 1/2$) and $\Omega_c = 1/2$ resonances (at 55.9 and 57.7 eV, respectively), have relatively small contributions from the nearby blue peaks, so the error in dephasing times induced by this overlap is within experimental uncertainty. The dephasing times are obtained directly from the fits. In the gas phase, the dephasing time differs from the core-excited states lifetimes only by a factor of two which accounts for the difference in probing a coherence rather than a population.[7] This yields lifetimes of 3.5±0.4 and 4.3±0.4 fs for the ($\Omega_c = 3/2,\ 1/2$) and $\Omega_c = 1/2$ states in the orange box of Fig. 1c, respectively.

For the blue peaks, corresponding to the $\Omega_c = 5/2,\ 3/2$ states at 55.6 and 57.4 eV, overlap contributions at these energies are much larger than the kinetic fit error bars, so the kinetic traces will have significant contributions from both states. Using the fit of the absorption spectrum in Fig S2c, contributions at 55.6 and 57.4 eV of nearby peaks are known. The peak centered at 55.9 eV decreases to 32 % of its intensity at 55.6 eV while the peak centered at 57.7 eV contributes 34 % of the maximum intensity at 57.4 eV.

The kinetic traces measured at 55.6 eV and 57.4 eV are corrected by subtracting the traces measured at 55.9 and 57.7 eV and scaled by their intensity decreases, i.e. 0.32 and 0.34, respectively. The subtracted kinetic traces are shown in Fig. S3a and b.

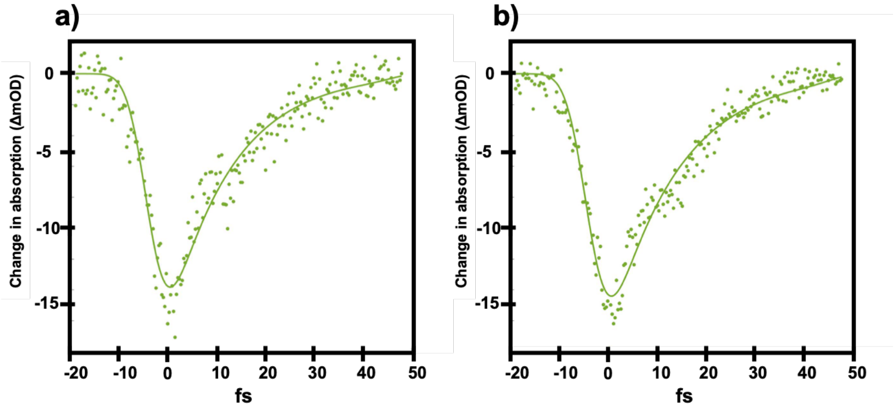

Figure S3a): Kinetic trace at 55.6 eV with the contribution of the trace at 55.9 eV scaled by 0.32 and subtracted in dots. The fit is shown as a solid line and yields a time constant of 12.9 ± 1.2 fs **b)**: Kinetic trace at 57.4 eV with the contribution of the trace at 57.7 eV scaled by 0.34 and subtracted. The fit is shown as a solid line and yields a time constant of 13.8 ± 1.2 fs.

The resulting traces are fitted using the function described earlier and yield dephasing times of 12.9 ± 1.2 and 13.8 ± 1.2 fs for the $\Omega_c = 5/2$ and $\Omega_c = 3/2$ core-holes, respectively. Accounting for the factor of two between dephasing times and lifetimes described earlier in the text[7] this yield lifetimes of 6.5 ± 0.6 and 6.9 ± 0.6 for the $\Omega_c = 5/2$ and $\Omega_c = 3/2$ core-holes in the blue box of Fig 1c respectively. Table S3 summarizes the various results.

Table S3: *Iodine 4d^{-1}6p CESs lifetimes in ICl*

Hole orientation	Perpendicular	Parallel	Perpendicular	Parallel
Core-hole character	$\Omega_c = 5/2$	$\Omega_c = 3/2, \quad 1/2$	$\Omega_c = 3/2$	$\Omega_c = 1/2$
Energy (eV)	55.6	55.9	57.4	57.7
Dephasing time (fs)	12.9 ± 1.2 *(from deconvolution)*	7.0 ± 0.8 fs *(from direct fit of ATAS data)*	13.8 ± 1.2 fs *(from deconvolution)*	8.6 ± 0.6 fs *(from direct fit of ATAS data)*
Lifetime (fs)	6.5 ± 0.6	3.5 ± 0.4	6.9 ± 0.6	4.3 ± 0.4

SM3: Core-orbitals with spin-orbit coupling

The core-orbital electronic densities are obtained by SCF calculations using infinite-order two-component relativistic Hamiltonian using DIRAC program package.[8] The core-orbitals electronic densities are obtained for the core ionized species. The isosurfaces of the electronic densities for a value of 0.03 (3 % of the electronic density) are shown in Fig. S4. Hund's case (c) notation is used due to the large spin-orbit interaction.[9] The states are designated by the labeling of the core-orbital character using the quantum number Ω_c. The orbital obtained including the spin-orbit interaction result from a mixing of the spin-orbit free orbitals (d_{z^2}, d_{xz}, d_{yz}, $d_{x^2-y^2}$ and d_{xy}) which are responsible for the different orbital' orientations observed. Fig S4 shows the main components of the orbitals with spin-orbit coupling.

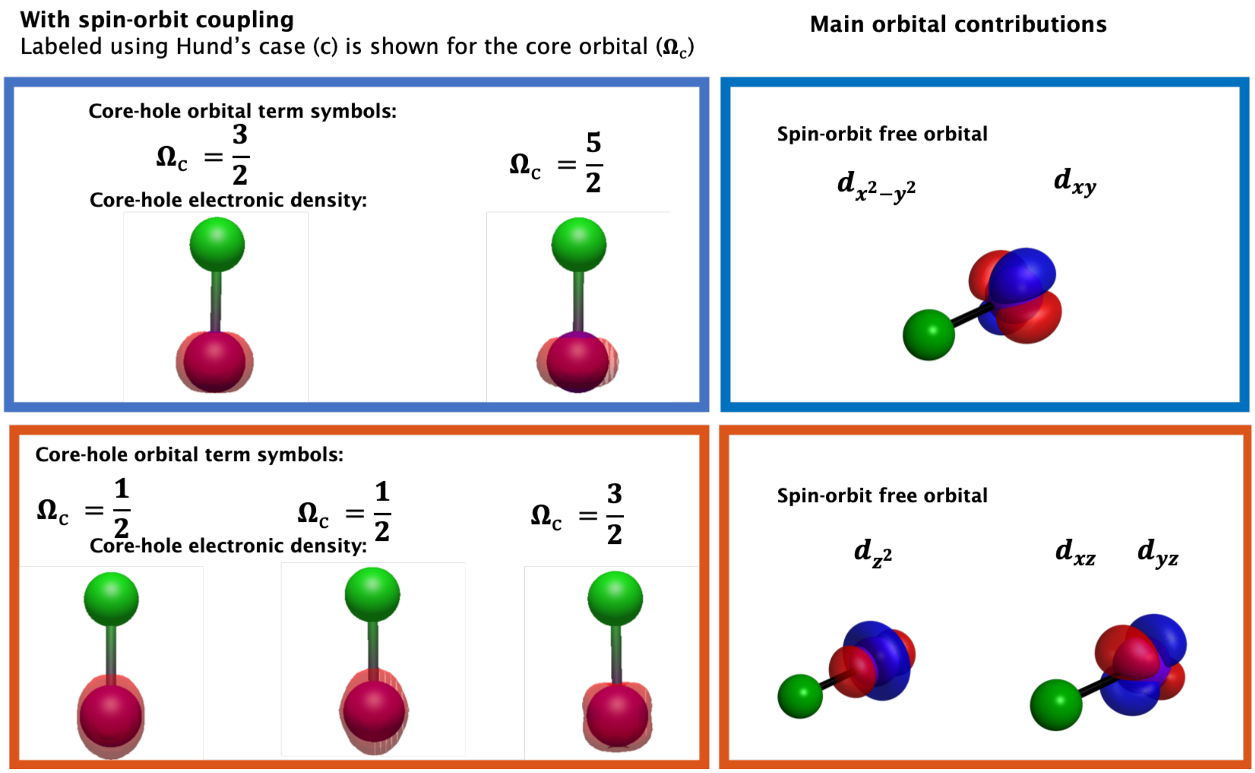

Figure S4: Core orbitals electronic densities obtained including spin-orbit coupling on the left and the wavefunction of the corresponding main components on the right. The quantum number Ω_c on the left hand side follows the Hund case (c).[10]

Decompositions of the orbitals are shown below where $|0\rangle$, $|1\rangle$ and $|2\rangle$ refer to (d_{z^2}), (d_{xz}, d_{yz}) and $(d_{x^2-y^2}, d_{xy})$ respectively while $|\alpha\rangle$ and $|\beta\rangle$ correspond to the two projections of the electronic spin on the internuclear axis.[10] The decompositions are consistent with previously published results.[11] The energies shown are the $4d \rightarrow 6p$ transition energies obtained from the absorption spectrum (c.f. SM1).

***Table S4:** Decompositions of the core-hole orbitals incluing the spin-orbit coupling interaction*

**Term**	**$4d \rightarrow 6p$ (in eV)**	**Eigenfunctions**
$\boldsymbol{\Omega_c = 5/2}$	55.6	$\vert 2\alpha\rangle$
$\boldsymbol{\Omega_c = 3/2}$	55.9	$\sqrt{0.2}\vert 2\beta\rangle + \sqrt{0.8}\vert 1\alpha\rangle$
$\boldsymbol{\Omega_c = 1/2}$	55.9	$\sqrt{0.4}\vert 1\beta\rangle + \sqrt{0.6}\vert 0\alpha\rangle$

$\mathbf{\Omega_c = 3/2}$	57.4	$\sqrt{0.8}\lvert 2\beta\rangle - \sqrt{0.2}\lvert 1\alpha\rangle$
$\mathbf{\Omega_c = 1/2}$	57.7	$\sqrt{0.6}\lvert 1\beta\rangle - \sqrt{0.4}\lvert 0\alpha\rangle$

SM4: Decay channels

As discussed in the methods section, the linewidth calculations make two assumptions, the core-excited cation is considered rather than the full core-excited state and relativistic effects are not taken into account. Despite these approximations, the calculation reproduces the trend in lifetime variations with the orientation of core-hole wavefunctions.

According to the calculations, forty-eight decay channels are accessible for the $I^+(4d^{-1})Cl$ initial state, which are composed of different combinations of two holes in the 7 valence MOs showed in Table S5.

Table S5 Valence MOs populated in the $I4d^{-1}$ decay. Shown are the orbital energies and the isosurfaces at R=2.4 Å

Decay product MO label	Energy (eV)	Isosurface
1	-10.76	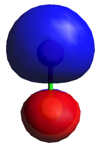
2	-10.76	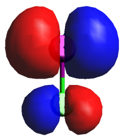

3	-13.68	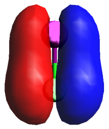
4	-13.68	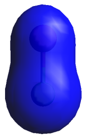
5	-14.14	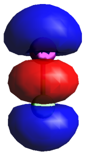
6	-24.61	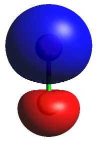
7	-30.05	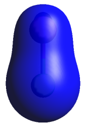

The forty-eight channels are obtained by projecting the two-hole combinations onto the two-hole-one-particle space, representing the final state and the outgoing electron.[12] The resulting two-hole configurations of the decay channels are shown in Table S6 for the main contributions. Each hole MO is indicated according to the label in Table S5 and the last letter is the spin state (s = singlet; t = triplet). The partial width of each channel is shown in Fig S5 for the three initial states considered.

Table S6 *Open decay channels*

Decay channels Number	Weight	Composition	Weight	Composition
1	0.798	MO_6_MO_7_t		
2	0.352	MO_6_MO_6_s	0.352	MO_7_MO_7_s
3	0.704	MO_6_MO_7_s		
4	0.114	MO_4_MO_7_s	0.114	MO_5_MO_6_s
5	0.499	MO_4_MO_6_s	0.499	MO_5_MO_7_s
6	0.455	MO_4_MO_6_t	0.455	MO_5_MO_7_t
7	0.455	MO_4_MO_7_t	0.455	MO_5_MO_6_t
8	0.499	MO_4_MO_7_t	0.499	MO_5_MO_6_t
9	0.841	MO_3_MO_6_t	0.116	MO_3_MO_7_t
10	0.116	MO_3_MO_6_t	0.841	MO_3_MO_7_t
11	0.359	MO_4_MO_6_t	0.359	MO_5_MO_7_t
12	0.877	MO_3_MO_6_s		
13	0.877	MO_3_MO_7_s		
14	0.301	MO_4_MO_6_s	0.301	MO_5_MO_7_s
15	0.301	MO_4_MO_7_s	0.301	MO_5_MO_6_s
16	0.290	MO_4_MO_7_s	0.290	MO_5_MO_6_s
17	0.900	MO_3_MO_5_t		
18	0.900	MO_3_MO_4_t		
19	0.847	MO_3_MO_5_s		
20	0.847	MO_3_MO_4_s		
21	0.939	MO_3_MO_3_s		
22	0.839	MO_4_MO_5_t		
23	0.140	MO_4_MO_4_s	0.536	MO_4_MO_5_s
24	0.268	MO_4_MO_4_s	0.280	MO_4_MO_5_s
25	0.385	MO_4_MO_4_s	0.385	MO_5_MO_5_s
26	0.618	MO_2_MO_6_t	0.276	MO_2_MO_7_t
27	0.276	MO_2_MO_6_t	0.618	MO_2_MO_7_t
28	0.560	MO_2_MO_5_s	0.264	MO_2_MO_6_s
29	0.560	MO_2_MO_4_s	0.264	MO_2_MO_7_s
30	0.855	MO_2_MO_5_t		
31	0.855	MO_2_MO_4_t		
32	0.988	MO_2_MO_3_t		
33	0.444	MO_1_MO_6_s	0.348	MO_2_MO_6_s
34	0.444	MO_1_MO_7_s	0.348	MO_2_MO_7_s
35	0.721	MO_1_MO_6_t		
36	0.721	MO_1_MO_7_t		

37	0.938	MO_2_MO_3_s		
38	0.362	MO_1_MO_6_s	0.247	MO_2_MO_5_s
39	0.362	MO_1_MO_7_s	0.247	MO_2_MO_4_s
40	0.975	MO_1_MO_3_t		
41	0.905	MO_1_MO_5_t		
42	0.905	MO_1_MO_4_t		
43	0.161	MO_1_MO_2_s	0.632	MO_1_MO_3_s
44	0.835	MO_1_MO_5_s	0.105	MO_1_MO_6_s
45	0.835	MO_1_MO_4_s	0.105	MO_1_MO_7_s
46	0.261	MO_1_MO_3_s	0.700	MO_2_MO_2_s
47	0.966	MO_1_MO_2_t		
48	0.755	MO_1_MO_2_s	0.103	MO_2_MO_2_s

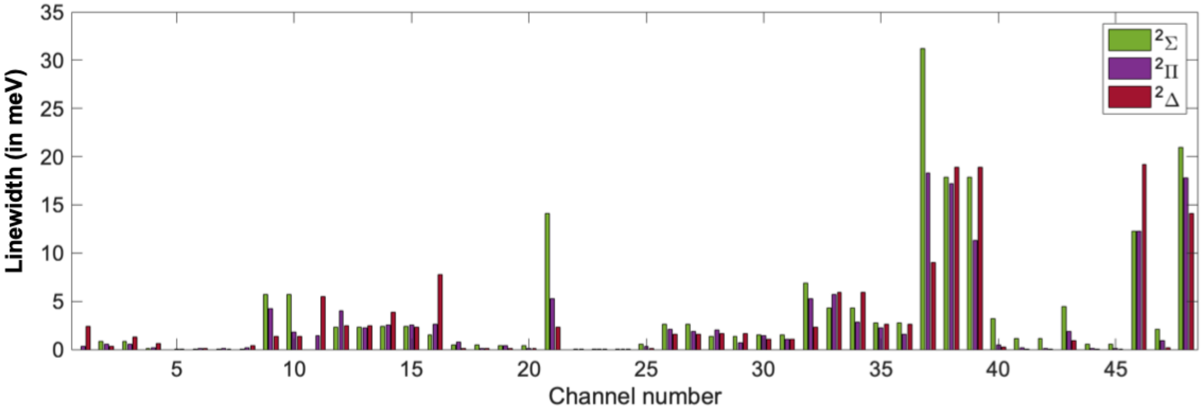

Figure S5 *open decay channels linewidths (in meV) at R=2.4 Å*

SM5: few cycle pulse characterization

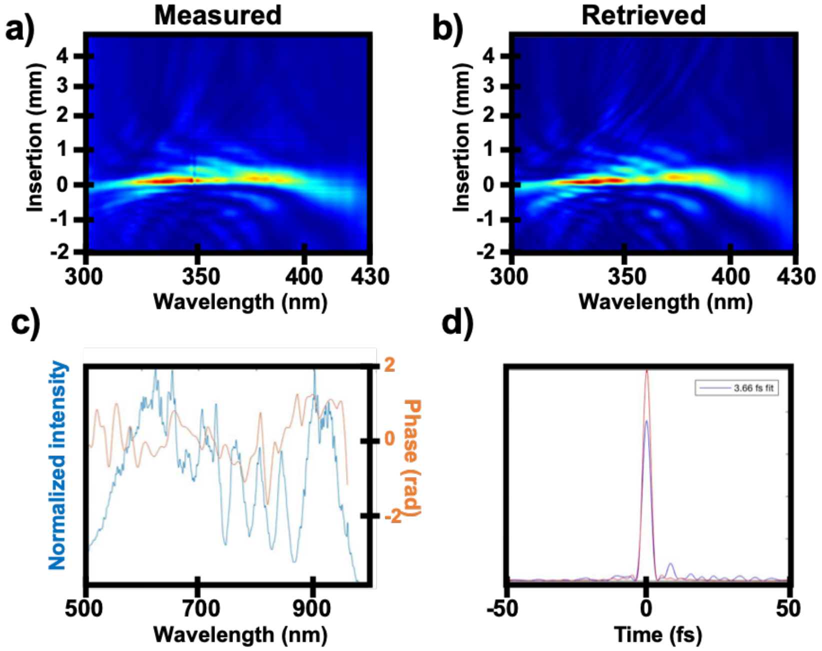

Figure S6: Characterization of NIR pulse used to drive high order harmonic generation and as probe beam uisng the d-scan technique in **a)** and **b)**, which shows, respectively, the measured and retrieved second harmonic spectrum for various wedge insertions. **c)** shows the linear spectrum (in blue) and spectral phase (in orange). **d)** shows the time profile of the pulse and the fit of a Gaussian pulse of 3.7 fs (FWHM, in red).

SM6: ATAS spectral resolution and power dependence

Spectral resolution effects

Determination of lifetimes by attosecond transient absorption (ATAS) relies on following the time evolution of the perturbed free induction decay resulting from the absorption of an XUV photon. The phase change imposed by the NIR pulse on the XUV free induction decay results in hyperbolic sidebands in the transient spectrum.[13] Due to finite spectral resolution, these sidebands can lead to an under-estimation of exited state lifetimes. It was shown in a previous study of inner valence excited states of xenon that accurate lifetimes could still be obtained with sufficient spectral resolution.[7] In those experiments a lifetime of 48.6 fs (FWHM of 13.5 meV) was measured with a spectrometer resolution of 10 meV (i.e. comparable to the FWHM of the resonance).

The ATAS experiment presented here was collected on the second order of the grating to optimize the spectral resolution to 50 meV. In analogy to the previous study of xenon, resonances with a comparable 50 meV FWHM can be measured, while longer lifetimes could be biased by the spectral resolution. This sets a lower limit of 50 meV on the state linewidth measurable without artefacts. As the state measured in ICl have linewidths > 80 meV, our spectral resolution does not bias our lifetime measurements. To confirm this, ATAS measurements were conducted on the core-transition of xenon at 65.1 eV and the lifetimes obtained in the time domain matched frequency domain measurements (detailed in SM7.)

In order to avoid reabsorption or resonant pulse propagation effects, the optical density of the sample was kept under 0.4, as studies have shown that non-linear third order spectroscopic experiments are free from lineshape distortion along as OD<1.2.[14]

Power dependence:

In ATAS experiments, the intensity of the NIR field affects the lineshapes of the transient features.[15] To ensure that the bleach recovery kinetics are not impacted by lineshape variations, traces are collected at two NIR peak power densities, 12 and 20 TW/cm^2, which are shown in Fig. S7 in red and black, respectively, for the four features discussed in this study.

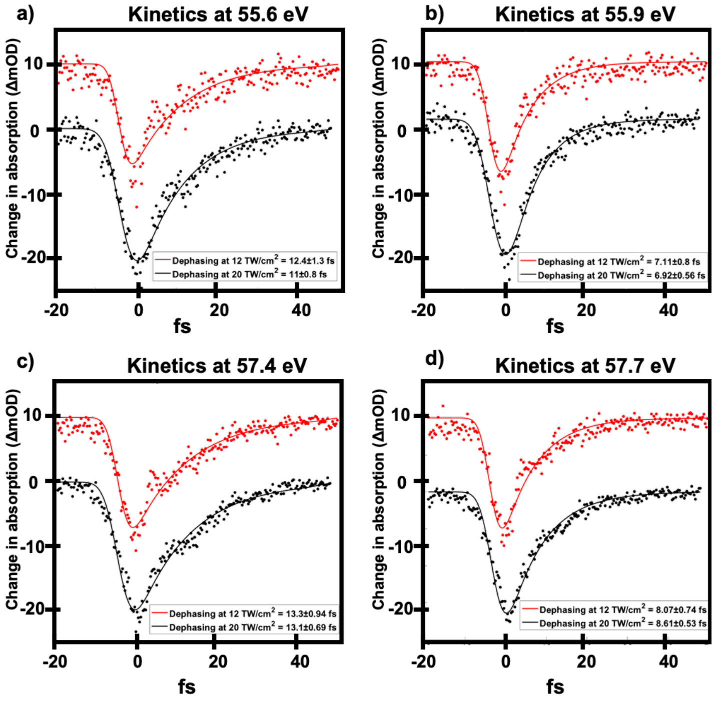

Figure S7: Kinetic traces of bleach recoveries at 12 and 20 TW/cm^2 for the four transitions discussed in the main text. The expriemental data are shown in dots and the fit in solid lines. The reported time constants are dephasing times, corresponding to twice the lifetime.

Averaging was increased for the transient spectrum at 12 TW/cm^2 in order to reduce the fit error bars. Kinetic fit results reported in Table S7 show no significant variation with NIR peak intensity and the dependence on the core-hole wavefunction alignment is still observed.

Table S7: Kinetic fits results at various NIR peak power density

Probed energy	**55.6 eV**	**55.9 eV**	**57.4 eV**	**55.7 eV**
At 12 TW/cm^2	10.2 ± 0.9 fs	8.1 ± 0.9 fs	12.5 ± 0.8 fs	7.6 ± 0.8fs
At 20 TW/cm^2	11.0 ± 0.8 fs	7.0 ± 0.8 fs	13.2± 0.6 fs	8.6± 0.6 fs

SM7: Lifetime measurement in Xenon:

To further confirm the accuracy of the experiment to measure lifetimes, a consistency experiment was conducted in xenon by measuring the lifetime of the $4d^{-1}6p$ core-excited state. This state was chosen because of its close similarity with the $4d^{-1}6p$ state in the iodine atom. Linewidth measurements of iodine and xenon atoms report sub-8 fs lifetimes in each case.[16],[17] The decays of the polarization will be twice these values.

The attosecond pulse spectrum covers simultaneously the iodine and xenon transitions at 55.5 and 65.1 eV, respectively, so the laser parameters are the same in both experiments. The ATAS spectrum of xenon is shown in Fig. S8a and the ground state bleach recovery with kinetics fit is shown in Fig. S8b.

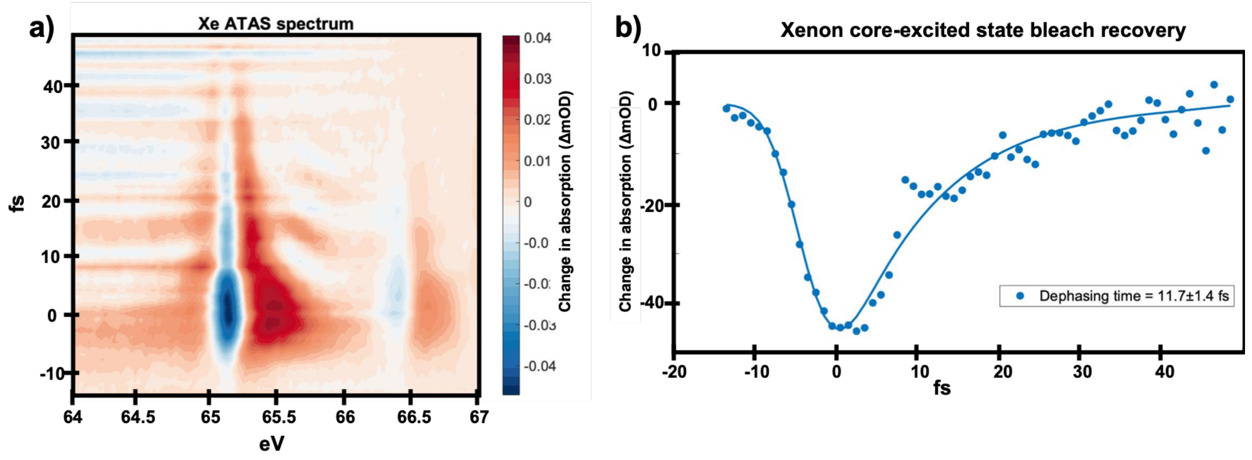

Figure S8: a) ATAS spectrum of xenon at the $4d^{-1}6p$ transition. b) Bleach recovery kinetics and fits of the $4d^{-1}6p$ transition

The bleach feature at 65.1 eV in the ATAS spectrum of Xe corresponds to the $4d^{-1}6p$ excited state. The bleach recovery fits to a 11.7 ± 1.4 fs dephasing time constant corresponding to a 5.9 ± 0.7 fs lifetime. Linewidth measurements of this transition in the literature yield a lifetime of 6.2 ± 0.2 fs,[17] confirming that the time domain measurement reports the correct lifetime within error bars.

References

(1) Kobayashi, Y.; Zeng, T.; Neumark, D. M.; Leone, S. R. Ab Initio Investigation of Br-3d Core-Excited States in HBr and HBr+ toward XUV Probing of Photochemical Dynamics. *Struct. Dyn.* **2019**, *6* (1), 14101. https://doi.org/10.1063/1.5085011.

(2) Zeng, T. A Diabatization Protocol That Includes Spin-Orbit Coupling. *J. Chem. Phys.* **2017**, *146* (14), 144103. https://doi.org/10.1063/1.4979902.

(3) Zeng, T.; Fedorov, D. G.; Klobukowski, M. Model Core Potentials for Studies of Scalar-Relativistic Effects and Spin-Orbit Coupling at Douglas–Kroll Level. I. Theory and Applications to Pb and Bi. *J. Chem. Phys.* **2009**, *131* (12), 124109. https://doi.org/10.1063/1.3211955.

(4) Schmidt, M. W.; Baldridge, K. K.; Boatz, J. A.; Elbert, S. T.; Gordon, M. S.; Jensen, J. H.; Koseki, S.; Matsunaga, N.; Nguyen, K. A.; Su, S.; et al. General Atomic and Molecular Electronic Structure System. *J. Comput. Chem.* **1993**, *14* (11), 1347–1363. https://doi.org/10.1002/jcc.540141112.

(5) Zeng, T.; Fedorov, D. G.; Klobukowski, M. Performance of Dynamically Weighted Multiconfiguration Self-Consistent Field and Spin-Orbit Coupling Calculations of Diatomic Molecules of Group 14 Elements. *J. Chem. Phys.* **2011**, *134* (2), 24108. https://doi.org/10.1063/1.3529840.

(6) V.A. Mozhayskiy and A.I. Krylov. EzSpectrum, Http://Iopenshell.Usc.Edu/Downloads. p http://iopenshell.usc.edu/downloads.

(7) Bernhardt, B.; Beck, A. R.; Li, X.; Warrick, E. R.; Bell, M. J.; Haxton, D. J.; McCurdy, C. W.; Neumark, D. M.; Leone, S. R. High-Spectral-Resolution Attosecond Absorption Spectroscopy of Autoionization in Xenon. *Phys. Rev. A - At. Mol. Opt. Phys.* **2014**, *89*, 023408. https://doi.org/10.1103/PhysRevA.89.023408.

(8) Jensen, H. J.; Bast, R.; Saue, T.; Visscher, L.; Bakken, V.; Dyall, K.; Dubillard, S.; Ekström, U.; Eliav, E.; Enevoldsen, T.; et al. *DIRAC19. DIRAC, a Relativistic Ab Initio Electronic Structure Program, Release DIRAC16 (2019)*; 2019.

(9) Lefebvre-Brion, H.; Giusti-Suzor, A.; Raşeev, G. Theoretical Study of the Spin-orbit Autoionization in Molecules Application to the HI Photoionization Spectrum. *J. Chem. Phys.* **1985**, *83* (4), 1557–1566. https://doi.org/10.1063/1.449392.

(10) Johnson, J.; Cutler, J. N.; Bancroft, G. M.; Hu, Y. F.; Tan, K. H. High-Resolution Photoabsorption and Photoelectron Spectra of Bromine-Containing Molecules at the Br 3d Edge: The Importance of Ligand Field Splitting. *J. Phys. B At. Mol. Opt. Phys.* **1997**, *30* (21), 4899. https://doi.org/10.1088/0953-4075/30/21/024.

(11) Cutler, J. N.; Bancroft, G. M.; Tan, K. H. Ligand-Field Splittings and Core-Level Linewidths in I 4d Photoelectron Spectra of Iodine Molecules. *J. Chem. Phys.* **1992**, *97*, 7932. https://doi.org/10.1063/1.463468.

(12) Ghosh, A.; Cederbaum, L. S.; Gokhberg, K. Electron Transfer Mediated Decay in HeLi2 Cluster: Potential Energy Surfaces and Decay Widths. *J. Chem. Phys.* **2019**, *150* (16), 164309. https://doi.org/10.1063/1.5082952.

(13) Wu, M.; Chen, S.; Camp, S.; Schafer, K. J.; Gaarde, M. B. Theory of Strong-Field Attosecond Transient Absorption. *J. Phys. B At. Mol. Opt. Phys.* **2016**, *49* (6), 062003. https://doi.org/10.1088/0953-4075/49/6/062003.

(14) Xiong, W.; Strasfeld, D. B.; Shim, S.-H.; Zanni, M. T. Automated 2D IR Spectrometer Mitigates the Influence of High Optical Densities. *Vib. Spectrosc.* **2009**, *50* (1), 136–142.

https://doi.org/https://doi.org/10.1016/j.vibspec.2008.10.010.
(15) Ott, C.; Kaldun, A.; Raith, P.; Meyer, K.; Laux, M.; Evers, J.; Keitel, C. H.; Greene, C. H.; Pfeifer, T. Lorentz Meets Fano in Spectral Line Shapes: A Universal Phase and Its Laser Control. *Science (80-.).* **2013**, *340* (6133), 716–720. https://doi.org/10.1126/science.1234407.
(16) Nahon, L.; Morin, P. Experimental Study of Rydberg States Excited from the d Shell of Atomic Bromine and Iodine. *Phys. Rev. A* **1992**, *45*, 2887. https://doi.org/10.1103/PhysRevA.45.2887.
(17) Penent, F.; Palaudoux, J.; Lablanquie, P.; Andric, L.; Feifel, R.; Eland, J. H. D. Multielectron Spectroscopy: The Xenon 4d Hole Double Auger Decay. *Phys. Rev. Lett.* **2005**, *95* (8), 83002. https://doi.org/10.1103/PhysRevLett.95.083002.
